# Molecular and Pathological Features of Paediatric High-Grade Gliomas

**DOI:** 10.3390/ijms25158498

**Published:** 2024-08-03

**Authors:** Luis Blasco-Santana, Isabel Colmenero

**Affiliations:** Pathology Department, Hospital Infantil Universitario del Niño Jesús, Avenida de Menéndez Pelayo, 65, 28009 Madrid, Spain

**Keywords:** glioblastoma, glioma, paediatric glioma, brain tumour, histone 3, H3K27, H3G34

## Abstract

Paediatric high-grade gliomas are among the most common malignancies found in children. Despite morphological similarities to their adult counterparts, there are profound biological and molecular differences. Furthermore, and thanks to molecular biology, the diagnostic pathology of paediatric high-grade gliomas has experimented a dramatic shift towards molecular classification, with important prognostic implications, as is appropriately reflected in both the current WHO Classification of Tumours of the Central Nervous System and the WHO Classification of Paediatric Tumours. Emphasis is placed on histone 3, IDH1, and IDH2 alterations, and on Receptor of Tyrosine Kinase fusions. In this review we present the current diagnostic categories from the diagnostic pathology perspective including molecular features.

## 1. Introduction

Brain and other central nervous system (CNS) tumours are the most common solid tumour, the most common solid cancer, and the greatest contributor to cancer death in children and adolescents aged 0–19 years [1]. Gliomas represent 40.9% of all brain and other CNS tumours, of which high-grade glioma represent 9.3% of the total [1]. Although other CNS neoplasms, like embryonal tumours, are potent contributors to paediatric mortality, paediatric high-grade gliomas (pHGGs) remain an important cause of death, with low 5- and 10-year survival [1]. Strikingly, up to 60% of high-grade gliomas are located in the brainstem [1]. Furthermore, pHGGs are a complex family of tumours biologically distinct from their adult counterparts [2,3]. This makes pHGGs an especially relevant group for investigation.

Currently, the term “glioblastoma”, formerly widely used for high-grade glial neoplasms, is now reserved for the diagnosis of “IDH-wildtype glioblastoma”, a CNS WHO grade 4 adult-type diffuse glioma characterised by the absence of alterations in IDH and H3 genes and specific histological or molecular alterations [4]. Although this paper is published in a Special Issue titled “Glioblastoma: State of the Art and Future Trends”, there are deep biological differences between pHGGs and IDH-wildtype glioblastomas, and they should not be confused.

The group of pHGGs represents a mixture of different neoplasms, all of which bear their own architectural and cytological variability under the microscope, also showing huge morphological overlap. Sturm et al. [5] demonstrated using methylation profiling that high-grade brain tumours showing the former Central Nervous System Primitive Neuroectodermal Tumour (CNS-PNET) histology were, in fact, a combination of tumours of different lineages. Some CNS-PNETs were reclassified as HGGs, and some of them were histone-3 (H3)-altered or belonged to the current MYCN subgroup of pHGG gliomas, H3- and IDH-wildtype. Furthermore, the authors found robust neuronal antigen expression in some HGGs, arguing that morphology and immunohistochemistry (IHC) alone may be limited in classifying highly malignant undifferentiated neoplasms [5]. 

Globally, divergences of prognosis among diffuse pHGG patients are better explained by tumour location and typical entity-defining molecular alterations than by histological features of malignancy [2,3,6,7]. Regarding tumour location, hemispheric tumours tend to have better survival (median overall survival 18.0 months; 2-year overall survival 32%) than midline tumours (median overall survival 13.5 months; 2-year overall survival 21.4%), while the worst prognosis is found in pontine tumours (median overall survival 10.8 months; 2-year overall survival 5.2%) [2]. From a molecular point of view, H3-altered tumours have a worse prognosis than H3-wildtype tumours [2,3]. 

**Figure 1:**
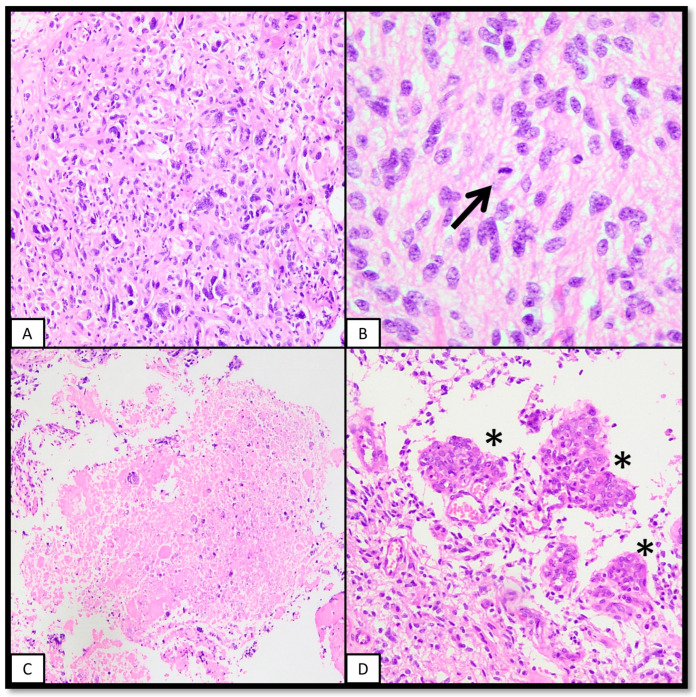
Microscopic features of malignancy in gliomas. (**A**): Cytological atypia, in the form of hyperchromasia, cytomegalia, irregularities in the nuclear membrane, etc, are criteria supporting malignancy. The example shown in this panel has evident atypical features that can be appreciated even at low-power magnification. (**B**): The presence of mitotic figures (arrow), if found in sufficient quantity, may upgrade some gliomas. Although most paediatric high-grade gliomas are per definition WHO grade 4 neoplasms, the presence of mitotic figures is supportive of its malignant behaviour. (**C**): Necrosis, with or without pseudopalisading, is indicative of a high-grade neoplasm. Although it does not define grade in paediatric high-grade gliomas, it suggests a highly malignant neoplasm. (**D**): Microvascular proliferation. The presence of vessels with multilayering of endothelium (asterisks) is a feature of aggressive neoplasms. Although not required in high-grade paediatric gliomas, it is concordant with an aggressive malignant glioma.

As will be shown later, most of the cases of pHGG are CNS WHO grade 4. The grade is assigned even in the absence of histological features of malignancy (brisk mitotic activity, microvascular proliferation, and necrosis with or without pseudopalisading; Figure 1) as long as entity-defining molecular alterations are present [4]. Furthermore, the contrary may also be true: necrosis or microvascular proliferation may not correlate with a worse prognosis [8].

These difficulties in providing a precise diagnosis based purely on morphology and immunophenotype entail profound diagnostic, prognostic, and therapeutic implications, and justify the current focus on the classification of the molecular pathology of CNS tumours [4].

Finally, accounting for a potential excess of molecular information yielded by massive genomic sequencing techniques, the current WHO Classification of Tumours of the CNS recommends multilayered, integrated diagnoses. These will include histopathological classification, WHO grade, and molecular information in tiers, allowing for the better classification of patients [4].

In this paper, we will review the clinical, radiological, histopathological and molecular features defining the different subtypes of pHGG.

A Comment on Notation of H3 Point Mutations

There are historical differences in the notation of point mutations in H3 histones. Initial papers disregarded the first amino acid, a methionine, as it was cleaved in an early post-translational state, and thus, not initially detected. This results in H3K28 or H3G35 point mutations being noted as H3K27 or H3G34, respectively. This discrepancy is particularly problematic at sites where two identical amino acids follow each other in the protein sequence, which happens (not only) in positions 34 and 35 of the H3.3 gene, both being a glycine. Current notations take into account this correction and reflect it accordingly (as H3K28M or H3G35R/V) [9,10]. 

For clarity, all point mutations will mostly be presented in their traditional notation except in tumour WHO definitions.

## 2. Diffuse Midline Glioma, H3K27-Altered

### 2.1. Definition and Grade

Diffuse midline glioma (DMG), H3K27-altered is an infiltrative midline glioma with the loss of H3 p.K28me3 and usually either an H3 c.83A>T p. K28M substitution in one of the histone H3 isoforms, an aberrant overexpression of EZHIP, or an EGFR mutation [4].

It is assigned a CNS WHO grade of 4.

Although cases with alterations in EGFR have the same affinity for the midline and concomitant loss of H3K28 trimethylation, they usually appear with a different clinical presentation [11] (please see Section 2.9). 

### 2.2. Molecular Pathology

Diffuse midline gliomas, H3K27-altered, may mutate either the canonical (replication-dependent) histones H3.1 (HIST1H3B and HIST1H3C) and H3.2 (HIST2H3C), or the non-canonical, replication-independent variant H3.3 (H3F3A) [2,12]. The most affected histone is the non-canonical replication-independent variant H3.3 (H3F3A), with over 80% of cases in some series [2,13,14]. H3.1-mutated cases make up the rest of the series, with only extremely rare cases bearing H3.2 mutations [2,14]. The affected residue, lysine 28, is conserved among all H3 variants [15].

H3K27M mutations in the H3 family of histones result in the global reduction in H3K27me3 [15,16,17]. However, as in most cases, the presence of a mutant H3 only contributes to a fraction of the total histone 3 pool (around 3–17%), and the global reduction in H3K27me3 marks is more extended than what would be expected by the mere loss of a methylation site due to the presence of a H3K27M mutation alone [17]. This effect on global H3K27 trimethylation may be explained by the interaction of H3K27-mutant histones with the Polycomb Repressor Complex 2 (PRC2).

PRC2 and its subunits EZH2 and SUZ12 are the enzymatic complexes responsible for the trimethylation of the H3K27residues [18]. The automethylation of PRC2 results in higher methyltransferase activity, essential for the conversion of H3K27me2 to H3K27me3 and the maintenance of H3K27me3 levels [18]. The exposure of PRC2 to H3K27M-mutant histones may induce a conformational change in the protein, resulting in a hypomethylated and hypoactive state of PRC2 [18]. Thus, impaired PRC2 automethylation results in a global reduction in H3K27me3 marks [18]. There is also a reduction in H3K27me2 marks, but minor when compared to the H3K27me3 reduction [17]. This results in a dominant inhibitory effect of the H3K27M mutant on the EZH2 and SUZ12 subunits of the Polycomb Repressor Complex 2 (PRC2), which profoundly disrupts cell homeostasis, with global a reduction in H3K27me3 and the presence of a DNA hypomethylation phenotype [15,16,17,19,20,21,22].

Further studies suggest that along with the global reduction in H3K27me3 marks, there is also a redistribution of these. In chromatin immunoprecipitation assays, the distribution of H3K28me3 marks is similar to the pattern of embryonic stem cells, with sharp peaks of H3K28me3 and the loss of broad H3K27me3 domains that would be found in normal somatic cells [17]. This redistribution of H3K27me3 marks is theorised to be driven by an impaired chromatin spread of PRC2 from its recruiting sites and reduced trimethylation enzymatic capabilities after the exposure of PRC2 to the H3K27M-mutant H3 histone [17]. 

There is also a reduced interaction of H3.3 K27M with H3K9 methyltransferases and methylases, resulting in a global reduction in H3K9 methylation marks [23].

EZHIP, an inhibitor of PRC2, is another pathway of gliomagenesis, via EZHIP overexpression with a concomitant loss of H3K27 trimethylation [24]. In t-distributed stochastic neighbour embedding (t-SNE) analysis, H3K27-altered gliomas and EZHIP-overexpressed gliomas cluster together, arguing in favour of them being the same functional entity [24]. 

H3K27-alterations represent the earliest tumorigenic event, since in tumour homogeneity studies they are equally distributed along the tumour mass [25]. This alteration is essential in the early stages of tumour development and is needed to preserve the proliferative and invasive potential [17]. However, supporting mutations are needed [26] and typically co-segregate depending on the type of histone 3 altered. On the one hand, H3.3-altered cases are associated with alterations in FGFR1, TP53, or PPM1D (which are mutually exclusive with TP53 alterations) [2,12,14,22,25,27]. The amplification of PDGFRA is also found more frequently in H3.3-altered gliomas [12,14]. Further evidence supporting PDGF pathway collaboration comes from murine models, where the co-occurrence of the H3.3 K27M mutation and PDGF-B overexpression increase cell proliferation and tumour grade [26]. H3.3-altered gliomas may also have mutually exclusive alterations in ATRX or TOP3A implicated in the Alternative Lengthening of Telomeres phenotype [2,12,27]. On the other hand, H3.1-altered cases are associated with alterations in ACVR1 [2,12,14,22,25,27,28]. ACVR1 and FGFR1 are mutually exclusive with TP53 mutations [27]. In rare cases, there is an association of H3.1 K27M mutation with PI3KR1 or PIK3CA alterations [22,25].

This clear segregation of mutations according to the type of histone altered may be explained by the cellular role of the histone in cell biology: Canonical histones H3.1 and H3.2 are synthesised during replication, and as such, mutations of ACVR1, a growth factor receptor that favours cell division, can better perpetuate pathological H3 synthesis. On the other hand, the non-canonical histone H3.3, which is not linked to replication, may depend more on evasion from TP53 genomic vigilance to deeply reshape the epigenomic landscape of the cell, driving tumour formation [25]. 

Furthermore, an analysis of gene enrichment profiling with clinical–radiological and histological correlation of two cohorts of H3.1- and H3.3-altered gliomas found differences in both tumours [12]. H3.3-altered gliomas were enriched in neural, oligodendrocytic, and proneural–glioblastoma multiform pathways. Additionally, genes implicated in the inhibition of metastasis were downregulated, arguing for the higher incidence of metastatic disease in these patients, as all but one of the fifteen patients with metastasis relapse corresponded to the H3.3 cohort [12]. On the other hand, H3.1-altered gliomas were enriched in genes of the astroglial cell and mesenchymal glioblastoma subtype signatures. Genes implicated in angiogenesis and hypoxia were also upregulated, suggesting a possible higher incidence of tumoral necrosis and extracellular oedema in this group. HIF1A activation was predicted as an underlying mechanism. Histological and magnetic resonance imaging (MRI) analysis of both cohorts showed more extracellular oedema and extracellular vacuoles in the H3.1 subgroup. Similar differences were found in the apparent diffusion coefficients (ADCs) and distributed diffusion coefficients (DDCs) in diffusion-weighted MRI, which argued for differential water distribution in both tumours [12]. 

RNA-seq analyses have further defined four cellular programs in diffuse midline glioma, H3K27-altered: cell cycle, astrocytic differentiation, oligodendrocytic differentiation, and oligodendrocyte precursor cells (OPC-like). OPC-like cells are implicated in self-renewal, propagation, and differentiation into other cellular programs. OPC-like cells are at least sustained by PDGRFA amplification, which are enriched in PRC2 target genes [19]. As such, PDGFRA may be a target for therapeutic schemes [19].

The top pathways altered in H3.3 K27M gliomas correspond to molecular and cellular functions, with an increase in cell-to-cell signalling and a decrease in cell cycle progression. The cellular functions affected by altered methylation in H3.3-K27M-mutated cells include embryonic development, decreased cell growth and proliferation, and increased cell-to-cell signalling [14]. 

Gene ontology analysis found that the upregulated genes are related to neural differentiation and developmental pathways [17]. There is also genomic co-enrichment of H3K27 acetylation marks [20]. This results in the low-level upregulation of many genes (many related to neurogenesis and stem cell features) and the downregulation of a few [17]. the upregulated genes tend to be enriched in PRC2 targets [19]. The ID1-ID4 family of genes [12,17] and OLIG2 [15] are among the upregulated genes, while p16Ink4A (CDKN2A) is among the downregulated ones [15]. There is also an upregulation of the subunit BMI1 of PRC1, probably representing a compensatory mechanism for the suppression of PRC2 [19]. 

Other studies support the increase in ID1 and ID2 expression levels in H3K27-altered gliomas, either through mutations in ACVR1 and the upregulation of pSMAD1/5 in H3.1-altered gliomas [14,27] or through other mechanisms in H3.3-altered gliomas [14]. ID1 is most expressed in glioma cells with the oligo/astrocytic precursor cell program [29]. Higher levels of expression of ID1 are also found in cycling cells when compared to non-cycling cells [29]. Globally, increased ID1 expression promotes the migration and invasion of tumour cells [29], contributing to the highly malignant character of this disease.

Regarding CDKN2A, a gene altered in a wide array of gliomas, structural alterations are rarely found in H3K27-altered gliomas [26]. However, murine models of H3.3-altered gliomas showed the accumulation of H3K27me3 marks at the p16ink4A promoter gene (CDKN2A), with a concomitant reduction in p16, supporting the notion that epigenetic mechanisms may play a role [26]. Interestingly, the inhibition of EZH2 activity through enzymatic inhibition did not restore p16 levels in H3.3 K27M murine tumours lines, even though a reduction in p16 H3K27me3 marks was achieved. This argues in favour of supplementary inhibitory mechanisms of p16 in H3.3 K27M glioma models [26]. In the same study, the inhibition of DNA methyltransferase activity with decitabine increased mRNA p16 levels and increased p16 protein expression in murine models and human Diffuse Intrinsic Pontine Glioma (DIPG) cell lines. These results suggest that DNA methylation also plays a role in H3K27-altered gliomas, especially in regulating p16 levels, which plays a major role in tumour development and proliferation [26].

As a final comment, although H3.1- and H3.3-altered gliomas both fall under the WHO umbrella term of diffuse midline glioma, H3K27-altered, differences in age of diagnosis, tumour location, length of survival, methylation pattern, gene ontology analysis, and typically associated gene mutations may argue that both entities, although similar, represent different diseases [30]. Unfortunately, although this approach is interesting, its application in glioma therapy still needs to be improved, as the prognosis is grim in both variants [2].

### 2.3. Epidemiology

This family of gliomas shows a variable spatial distribution along the midline depending on the type of H3 mutated and the age of the patient. Globally, in some paediatric glioma and glioblastoma cohorts, this family of tumours shows an incidence around 27–43% [2,3,6,31,32]. Although most patients are children and adolescents, with a median age of 14 years in some series [33], young adults may occasionally be affected. According to the type of H3 mutated, we see the following:

− H3.1/H3.2-altered cases are restricted to the pons [27], occur in younger patients (median age 5 years), and have slightly longer survival compared to the H3.3-altered counterpart (median 15.0 months) [2,12,32];
− H3.3-altered cases are distributed along the midline, occur in older patients, and have shorter survival (median 11.0 months) [2,32]. The midline structures affected comprise the pons, thalamus, spinal cord [34], third ventricle, hypothalamus, cerebellum, and pineal gland [2,33,35,36].

If we classify these tumours according to location, non-pontine locations are more common in adolescents and older children [31]. Patients with pontine gliomas tend to be younger (median 7 years), while the thalamic and spinal counterparts tend to occur in older patients (median 24 and 25 years, respectively) [33]. Spinal cord cases tend to be older than the pontic or thalamic counterparts [34,37,38]. The thoracic segment is the most affected [34,37,38]. Most spinal cases will bear the H3.3 K27M mutation [34,37,38], with exceptional cases showing rarer mutations, like H3F3A G34W, typical of the giant cell tumour of the bone [37], or H3F3B K27I [37]. 

Finally, although rarer, this tumour can also present in adults. It is still limited to midline structures, with a predominance of non-pontine locations (spinal cord, thalamus, brainstem, cerebellum, and pineal gland). Although the age range is extensive (18–82), patients tend to be younger, with a median age of 32. There is a preference for the spinal cord and thalamus. Most cases are H3.3-altered [36].

### 2.4. Imaging

A diffuse tumour residing in the midline is the typical presentation on imaging. Pontine cases require a diffuse involvement of at least 50% of the pons to qualify as Diffuse Intrinsic Pontine Glioma (DIPG) [39,40]. 

In their systematic review of the literature, Lasocki et al. [41] commented on the heterogeneity of studies published. Thalamic and brainstem (in particular the pons) are the most commonly affected locations [42]. The spinal cord is the third most commonly affected location. 

On MRI, tumours tend to be hyperintense in T2-weighted sequences and homogeneous in T1-weighted sequences [42]. No MRI imaging differences seemed to occur among H3.1- and H3.3-altered tumours [42]. Both well- and ill-defined tumoral margins may be identified. 

For pontine tumours, wrapping around the basilar artery may be an important diagnostic sign in favour of H3K27-altered tumours [43].

Extrapontine extension, necrosis, and ring enhancement may also be present [40].

Contrast enhancement is highly variable, ranging from a lack of enhancement to ring enhancement with central necrosis [41,44,45]. Tumoral haemorrhage shows similar variability and seems to have little predictive value [41].

ADC values show a recurring trend in H3K27-altered gliomas towards lower values [41,46]. Chen et al. [47] showed in their series that H3K27-altered gliomas had lower values of minimal ADC, peritumoral ADC, and its corresponding ratios when compared to H3 wildtype gliomas. Thust et al. [46] also commented on ADC values showing variability to a certain degree. There is differing evidence regarding ADC values in H3.1-altered gliomas compared to H3.3-altered cases. Some series found lower ADC values [12], while others found the contrary [48]. H3.1-altered cases also seem to have lower perfusion values than the H3.3-altered counterpart [48].

Patients may develop leptomeningeal spread [39]. Dissemination may occur, generally as a later event during disease progression [41].

Cases in adults can show similar radiological features on MRI, with variable contrast enhancement [45] and blurred tumour shape in most cases. Non-pontine locations are more frequent, with the spinal cord and thalamus comprising more than 50% of cases in two case series [36,45]. Spinal cases may show an expansile mass [37].

### 2.5. Pathology

Diffuse midline gliomas, H3K27-altered, show a diffuse pattern of infiltration and classic astrocytic morphology (ovoid to elongate nuclei, coarse chromatin, etc), at least focally. Features of anaplasia, like brisk mitotic activity, necrosis, and microvascular proliferation, may be either present or absent [33,39]. DMGs are considered CNS WHO grade 4, irrespective of the presence of microvascular proliferation or necrosis. 

DMGs typically express OLIG2, MAP2, and S100. GFAP is variable, except for the EGFR-mutant subtype, which is typically GFAP-positive but may lack OLIG2 and SOX10 (see Section 2.9). Neurofilaments and synaptophysin are negative in the tumour cells but may highlight the infiltrated neuropil in the background. There may also be intense positivity for p53 IHC, and a loss of ATRX nuclear expression [33].

**Figure 2:**
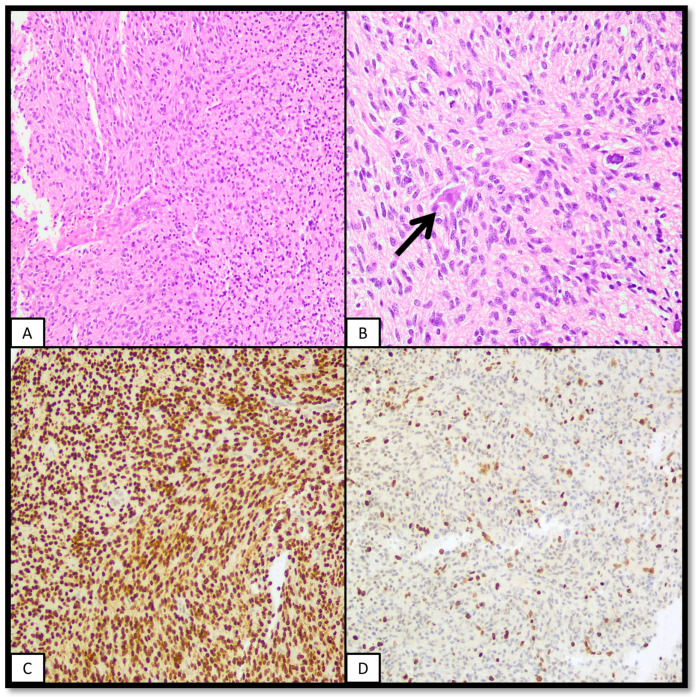
Diffuse midline glioma, H3K27-altered. The following figure features a glial neoplasm located in the midline of a paediatric patient. (**A**): Hypercellular neoplasm concordant with a glioma. (**B**): Presence of entrapped neurons (arrow) is supportive of its diffuse nature. (**C**): IHC for H3K27M shows diffuse and intense nuclear positivity. Note that vascular endothelia are negative, serving as an internal negative control. (**D**): IHC for H3K27me3 shows concomitant loss of or reduction in the marker in the neoplasm, with retained nuclear staining in entrapped normal elements, like neurons or endothelia.

There are IHC data available for both H3K27M (H3K28M) and H3K27me3 (H3K28me3) [12,21]. IHC positivity for the H3K27M antibody correlates well with H3K27M mutations [12,20,21,49]. H3.1-mutated cases may show a lower intensity of H3K27M IHC staining than H3.2- and H3.3-mutated cases [12]. Non-specific cytoplasmic staining may be a pitfall [33]. Other H3.3 mutations, like H3K27I, may need sequencing, as this mutation may not be recognised by IHC [12,37]. H3K27M nuclear positivity correlates with the reduction in H3K27me3 IHC expression [20]. Also, as not all tumours of this family bear the H3K27M mutation, it is still necessary to perform H3K27me3 IHC staining [4]. H3K27Ac IHC does not correlate well with H3K27M IHC positivity [20]. Ultimately, the loss of H3K27me3 staining plays a unifying role in diagnosing a molecularly varied entity [11]. A typical case of diffuse midline glioma, H3K27-altered, is featured in Figure 2.

H3K27M-mutant gliomas will also have low EZHIP expression levels and will be negative for EZHIP IHC [24]. On the other hand, EZHIP-altered gliomas will show a loss of H3K27me3 IHC, and will be negative for H3K27M IHC and positive for EZHIP IHC [24].

DMGs in the adult setting are similar to paediatric cases [36].

### 2.6. Differential Diagnosis

Any glioma occurring in the midline must include diffuse midline glioma, H3K27-altered, in the differential diagnosis, and as such, H3K27me3, H3K27M and EZHIP IHC need to be included in the diagnostic panel [20,21,24,33,50]. Caution is required when interpreting H3K27M and H3K27me3 IHC, as other non-diffuse midline gliomas may harbour an H3K27M mutation [49,51,52,53]. Furthermore, some CNS tumours may lose H3K27me3 (e.g., atypical teratoid/rhabdoid tumour), so a complete integrated diagnostic panel should be performed [21].

Other H3.3 mutations, like H3K27I, may need sequencing, as this mutation may not be recognised by IHC [12,37]. Since H3- and IDH-wildtype diffuse high-grade gliomas, MYCN subtype, may occur in midline structures, FISH for MYCN rearrangements would be recommended [50]. Diagnostic criteria [4,54] must be stringent to avoid the misdiagnosis of a low-grade tumour fortuitously having the H3K27M mutation [49] (see below, in 2.8 Non-diffuse H3K27-altered neuroepithelial tumours).

DMGs in the adult setting should also be considered when midline structures are affected and typical mutations of adult-type tumours are lacking. Patients also tend to be younger when compared with other adult-type gliomas, like IDH-mutant gliomas or IDH-wildtype glioblastomas [36].

Finally, although differential diagnosis may be complex due to a wide range of lesions that may also show a preference for the midline location, or mimic core molecular features (such as bearing H3K27 alterations with a concomitant loss of H3K27 trimethylation), WHO diagnostic criteria remain strict and unambiguous, requiring a glial (excluding embryonal neoplasms and other lineages), diffuse (excluding non-diffuse tumours [49,51,52,53]), midline, H3K27-altered (with H3K27 trimethylation loss and a concurrent H3 molecular alteration, such as H3K27M) neoplasm. Only if the neoplasm shows convergence of all these features may it qualify for the diagnosis of diffuse midline glioma, H3K27-altered [4] (see Section 2.10).

### 2.7. Prognosis

The prognosis in diffuse midline glioma, H3K27-altered, is dismal, with a median overall survival (OS) of 11 months and a 2-year survival around 5% [2]. Subtle differences in survival and response to treatment have been described among patients, mostly accounting to the type of mutated histone. Long-term survival (2 or more years of survival from diagnosis) is associated with H3.1 mutations [2,40]. The type of histone altered is a strong predictor for survival, with H3.1-altered cases being longer survivors [12]. Despite longer survival (median 15 months) in H3.1/H3.2-altered cases, the prognosis is equally grim [2,12,40]. Patients younger than 3 years and older than 10 years at diagnosis are more likely to be long-term survivors [40]. A regularly bad prognosis argues in favour of assigning a WHO grade 4 independent of histology [4]. Histological grade does not play a major role in patient survival [12,39]. EZHIP-altered gliomas bear a similar prognosis to H3K27M-mutant gliomas [24].

DMGs described in adults are equally discouraging, with a median survival between 10.4 and 19.7 months [36].

TP53 mutations may drive radioresistance [55]. H3.1-altered gliomas tend to have better responses to radiotherapy [12]. CHK1 inhibitors may increase sensitivity to radiotherapy [55]. p16 repression in H3.3-K27-altered glioma may increase sensitivity to CDK4/6 inhibition [26]. Murine models suggest that elevated levels of ID1 expression may be targetable with cannabidiol treatment [29].

Regarding the methylation status of the promoter of the MGMT gene (O^6^-methylguanine-DNA-methyltransferase, a known predictive factor of response to temozolomide in adult-type gliomas [56]), few cases show methylation and potential response to temozolomide. In their review of the literature, Vuong et al. [57] reported that 10.2% (25 cases of 245) of diffuse midline gliomas, H3K27-altered, showed methylation of the promoter. Paediatric cases tended to have a lower incidence of methylated promoters compared to adults: 6.1% (8 cases out of 131) versus 14.9% (17 cases out of 114) [57]. Along the same lines, Banan et al. [58] presented a series of 46 cases where none showed methylation of the MGMT gene promoter. Globally, it appears that methylation of the MGMT promoter is a rare event in diffuse midline glioma.

### 2.8. Non-Diffuse H3K27-Altered Neuroepithelial Tumours

H3K27M mutations have been reported in non-diffuse gliomas including subependymoma [53], posterior fossa group A ependymoma [52], ganglioglioma [51], anaplastic ganglioglioma, and pilocytic astrocytoma [49]. This compilation of H3K27M-mutation-positive cases [51,52,53] has some common aspects [49]. The histology is that of their corresponding entities, [51,52,53], and most cases have a predilection for midline structures [49,51,52,53]. There is a concomitant loss of H3K27me3 associated with H3K27M mutation [49,51,52,53]. Some tumour types have a combination of their typical molecular alterations, like BRAF V600E in ganglioglioma [51], to which H3K27M appears superimposed [49,51]. Some cases may undergo spontaneous malignant transformation [51]. The prognosis usually is not as bad as in typical instances of diffuse midline glioma, H3K27-altered [49,51,53,54]. However, some patients may have a worse prognosis when compared with their H3K27M-negative counterparts. Therefore, classifying these rare cases as WHO grade 4 based on an isolated mutation is probably an error. These facts reinforce the stringent and self-explanatory definition of diffuse midline glioma, H3K27-altered: it is a glioma with a diffuse histology, located in the midline, with H3K27 alterations [4,54].

Recently, these intriguing tumours were further studied by Auffret et al. [59] with integrated radiological, histopathological, genomic, transcriptomic, and methylation profiling. Most cases had prior diagnoses of pilocytic astrocytoma, grade 1–3 midline ganglioglioma, and diffuse gliomas, grade 4. Apart from some radiological (presence of macrocalcifications, circumscribed or nodular–diffuse aspect) and histological (ganglioma-like or piloid–HGG aspect, CD34 extravascular staining) differences, they also found that tumours with the co-occurrence of H3.3 K27M and BRAF or FGFR1 mutations tend to form their own cluster in unsupervised clustering methylation analysis, segregated from diffuse midline gliomas, H3K27-altered. Furthermore, these tumours tend to have, in gene set enrichment analysis, an enrichment in the MAPK and PI3K/AKT/MTOR pathways, also with an activation of senescence and p53 signalling. When compared with classic DMG, H3K27-altered, survival also tends to be longer (median OS of 37 and 36 months in BRAF-altered and FGFR1-altered H3K27M gliomas, versus 12 months of median OS in BRAF- and FGFR1-wildtype H3K27M gliomas). Accounting for all these differences, they propose the new category of diffuse midline glioma, H3K27 and BRAF/FGFR1 co-altered [59]. New prospective analyses will be needed to evaluate this new diagnostic category.

There is also the extremely rare setting of cortical diffuse gliomas with an H3K27M mutation, with the typical loss of H3K27 trimethylation. These cases are highly uncommon, and their prognosis and the optimal treatment regimens for these patients are not yet defined. These cases do not qualify as diffuse midline gliomas, H3K27-altered, and should not be assigned a WHO grade of 4 [60]. A Not Elsewhere Classified (NEC) diagnosis would be the best course of action [4].

### 2.9. Bithalamic Glioma (Diffuse Midline Glioma, EGFR-Altered)

This rare glioma presents as a symmetric, bilateral infiltration of both thalami [11,61,62]. 

Most tumours of this category have diverse alterations in EGFR, including in-frame insertions/duplications in exon 20, point mutations including a hotspot p.A289V/T substitution in exon 7, or amplifications [11,62]. Mutations in TP53 are also common [11,62]. Other reported molecular alterations include gains of chromosome 7, 1q [11,61,62], 5 or 5p [62], and the loss of chromosome 6q [11] or 17 [62]. Besides, this variant has its own methylation subgroup (separated from the H3K27-altered/EZHIP-overexpressed subgroup) in a t-SNE analysis enriched in pHGG, further reinforcing its value as an entity in itself [11,62]. 

The age at diagnosis is variable, usually with a wide age range; however, there is a clear paediatric predominance, with a median age at diagnosis of 8.2 years spanning an age range of 1 to 43 years [11]. 

Most cases are T2 and FLAIR hyperintense and a lack of or minimal enhancement after contrast infusion. Basal ganglia, brainstem, or insula can be infiltrated at diagnosis [62]. 

The histology is that of a diffuse astrocytoma [61], with or without features of anaplasia. This tumour is usually GFAP-positive but may lack OLIG2 and SOX10 [4]. Typically, although H3K27 mutations are lacking, there is a loss of H3K27me3 [11,61,62]. At least some cases show EZHIP overexpression, which may justify global H3K27me3 loss [11]. 

The differential diagnosis includes both low-grade (pilocytic astrocytoma) and high-grade gliomas that can affect thalami [11,61]. 

The prognosis is sombre, with a median OS of 8–12 months in paediatric patients, in line with a WHO grade 4 tumour [4,11,61,63]. Specific inhibitors may target EGFR alterations [62].

### 2.10. WHO Diagnostic Criteria

Current recommended criteria include:

- A diffuse cellular glioma;
- Loss of H3 p. K28me3 (K27me3 IHC);
- Midline location;
- One of the following:
  - Presence of an H3 p.K28M (K27M) or p.K28I (K27I) mutation (for H3K27-mutant subtypes);
  - Presence of a pathogenic mutation or amplification of EGFR (for the EGFR-mutant subtype);
  - Overexpression of EZHIP (for the H3-wildtype with EZHIP overexpression subtype);
  - Methylation profile of one of the subtypes of diffuse midline gliomas.

## 3. Diffuse Hemispheric Glioma, H3G34-Altered

### 3.1. Definition and Grade

This is an infiltrative glioma characterised by high-grade features (hypercellularity, mitosis, necrosis and/or microvascular proliferation), a hemispheric location, and the presence of a point mutation in G35R or G35V in H3F3A (H3.3) [4]. 

It is assigned a WHO grade of 4. 

### 3.2. Molecular Pathology

The H3.3 G34R mutation is more common (94%) than the H3.3 G34V mutation (6%) [64]. From a molecular perspective, the presence of a H3F3A G34R/V mutation disrupts the interaction of H3 with SETD2 and other methyltransferases by allosteric hindrance in cis [65,66,67,68,69], redistributing and reducing global H3K36 trimethylation marks [15]. Although other methyltransferases can methylate H3K36 residues, SETD2 is the only methyltransferase that can trimethylate H3K36 residues [67]. Interestingly, there is also a reduction in interaction with H3K9 methyltransferases and methylases, which results in a net increase in H3K9 trimethylation marks when compared with other pHHGs, especially with diffuse midline glioma, H3K27-altered, which is markedly reduced [23]. 

H3K36 methylation marks (H3K36me2 and H3K36me3) have deep local effects, antagonising PRC2 and ZMYND11 activity in cis [67,70]. On the one hand, unimpeded PRC2 activity, the consequence of a reduction in H3K36 methylation marks by G34 mutations results in an increase in H3K27me3 marks, which may lead to a global inhibitory tone in genes enriched in H3.3 histones [67]. As H3.3 histones tend to be located in promoters, gene bodies, and enhancers of genes with transcriptional activity, this can result in profound transcriptional disruption and immaturity [67]. On the other hand, ZMYND11 is implicated in transcriptional repression, acting as a modulator of elongation and the splicing of highly expressed genes. ZMYND11 shows reduced binding to H3.3 G34R histones, which in turn results in the transcriptional upregulation of gene programs that were actively repressed [70]. 

There is also global DNA hypomethylation across the whole genome, particularly in chromosome ends [71]. Reduced interaction of H3G34R with the DNA methyltransferases DNMT1 and DNMT3A may explain this global reduced DNA methylation [23]. However, despite global DNA hypomethylation, some genes show hypermethylation, such as MGMT, OLIG1, and OLIG2 [64,71]. 

The gene ontology analysis shows that differentially expressed genes in this tumour are involved in forebrain and cortex development [72]. Studies on forebrain and hindbrain foetal neural stem cells (NSC) found that H3G34 mutations have an oncogenic effect on forebrain NSC, but not on hindbrain NSC (in which a senescence phenotype was induced, via CDKN1A/p21), supporting the regional identity found in H3.3 point mutations in pHGG [70]. FOXG1, a transcription factor that acts as the master regulator of forebrain identity, has been proposed as an essential actor in these regional differences [70]. FOXG1 can act as a suppressor of CDKN1A/p21 and its mediated senescence, which provides an attractive explanation for some of the regional differences found in H3.3 mutations [70].

Furthermore, reduced global H3K36 methylation may hinder the H3K36me3-mediated mismatch repair (MMR) of the genome, mainly by reduced MutSα recruitment, resulting in higher mutation frequency and a mutator phenotype. However, this reduced MMR does not result in an actual microsatellite instability phenotype, as no microsatellites were found [66].

MYCN is especially enriched in this tumour, although no gene amplifications have been described [72]. One postulated mechanism for MYCN enrichment relies on FBXW7 deletion (located in chromosome 4q31.3; 4q loss is found in 70% of H3G34-altered gliomas) [64,73], which is a component of the SCF-like ubiquitin ligase complex that targets MYC/MYCN for proteasomal degradation [2,74]. The downregulation of FBXW7 leads to the accumulation of MYCN [74].

Other typical molecular alterations found in this family of tumours are mutations in TP53 (88%), ATRX (95%) [2,3], the amplification of PDGFRA (27%), 3q loss (67%), 4q loss (70%), and Alternative Lengthening of Telomeres (in line with the presence of ATRX mutations) [64,73]. A high proportion of patients (74%) also show methylation of the MGMT promoter [2,3,64]. There is also the enrichment of some alterations according to histology amplifications (morphology is commented in 3.5 Pathological features): The glioblastoma multiform-like (GBM-like) histology showed enrichment in PDGFRA and CDK6 amplifications, while the PNET-like histology showed a higher incidence of CCND2 [64].

It has yet to be elucidated how the presence of a point mutation in a translation-independent histone that usually represents 5% of the global H3 density can disrupt global genomic homeostasis at such a magnitude. H3K27 mutations have a dominant negative effect that hinders PRC2 functions locally and globally. However, the same effect is not found in H3G34 point mutations, where H3K36 methylation is impeded only in the histone carrying the mutation [75,76]. The findings of Bressan et al. [70] suggest that H3G34 point mutations have a local effect on cells with a vulnerable forebrain origin, in which the original progenitor state of the cells is recovered via the re-expression of pre-existing programs that were actively repressed [70]. 

Finally, Nguyen et al. [77] provide an excellent in-depth review of the molecular pathogenesis of diffuse hemispheric glioma, H3G34-altered.

### 3.3. Epidemiology

This tumour occurs predominantly in adolescents and young adults, with the vast majority being between 11 and 30 years (median: 18–19 years; range 9–52 years). There is a slight male predominance (1.4:1) [31,64,71]. In some paediatric glioblastoma cohorts, an incidence of around 15% has been reported [6].

### 3.4. Imaging Features

On MRI, diffuse hemispheric gliomas, H3G34-altered are hemispheric lesions with heterogeneous features. There is a preference for the temporal and parietal lobes [2,3,75,78,79]. Mass effect, contrast enhancement, necrosis, cystic components, haemorrhage, and calcification may be identified [79]. 

Furthermore, Picart et al. [78] found in their series of 17 cases that all tumours were hemispheric and monocentric at diagnosis. Midline extension could happen, but as an extension from an initially hemispheric tumour. Most cases were ill-defined, infiltrative cortico-subcortical neoplasms. Most cases showed areas of ADC restriction on diffusion-weighted imaging, with no or faint contrast enhancement. However, all cases that showed no or faint enhancement and underwent follow-up MRI showed new nodular or ring-like enhancements, with a median interval of 2.6 months [78].

Puntonet et al. [80] found similar features on their series of 12 patients. They reported that these tumours tended to be well-delineated, voluminous neoplasms, with subfalcine herniation in a high proportion of cases. Leptomeningeal contact was also the norm, found in all cases. Contrast enhancement and necrosis were variable. Peritumoral oedema was mild or absent. 

### 3.5. Pathological Features

Two different patterns are recognised:

- Approximately three quarters of tumours show a typical malignant glioma morphology (GBM-like), with hypercellularity, brisk mitotic activity, microvascular proliferation, and necrosis. Intense positivity for GFAP is usually found [64]. Some cases may show a dysplastic neuronal component, with bi- or multinucleation and the expression of CD34 [81];
- One quarter show a high-grade small-cell monomorphic proliferation reminiscent of embryonal tumours of the CNS (PNET-like histology). Structures resembling Homer–Wright rosettes may be identified. Intense positivity for synaptophysin and MAP2 is typical of this variant. Focal GFAP positivity may also be seen [64].

Shared features for both patterns are the lack of OLIG2 IHC staining, strong nuclear positivity for p53 protein (defined as >30% of tumour nuclei), and loss of nuclear ATRX protein, which should raise high suspicion for this entity [3,64,78,82]. In one case series, an OLIG2 IHC cut-off of 3% was proposed, with positivity below this value suggesting an H3G34-altered tumour [83]. 

**Figure 3:**
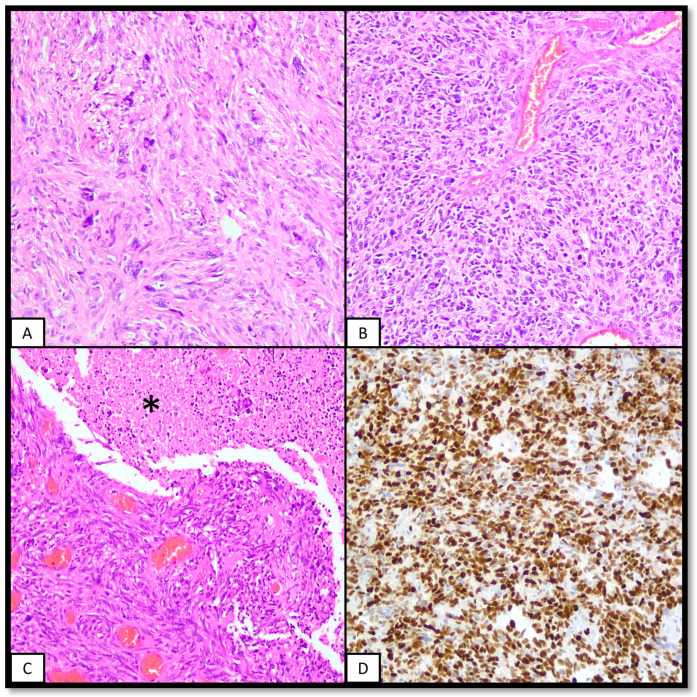
Diffuse hemispheric glioma, H3G34-altered. The following figure features a glial hemispheric neoplasm of an adolescent patient. (**A**,**B**): These two panels show a heterogeneous neoplasm, where glial features like eosinophilic cytoplasm and indistinct cellular borders (**A**) coexist with more embryonal/oligodendroglial features like a higher nucleus-to-cytoplasm ratio and round cell morphology (**B**). (**C**): High-grade features, like abundant mitoses, necrosis (asterisk), or microvascular proliferation are present. (**D**): IHC for p53 shows diffuse strong nuclear accumulation of the protein. There was also loss of ATRX and OLIG2 and concomitant lack of mutations in the IDH1 and 2 genes.

Although there are IHC data available for both H3G34R and H3G34V mutations, sensitivity and specificity are not perfect. Therefore, H3F3A sequencing remains the gold standard for molecular diagnosis [82]. Alterations in H3F3A and IDH1/2 are mutually exclusive; therefore, no H3K27 nor IDH1/2 alterations should be found [71]. Figure 3 features one case of diffuse hemispheric glioma, H3G34-altered.

### 3.6. Differential Diagnosis

As patients affected with this tumour tend to be older, differential diagnosis should also include IDH-mutant tumours [2,3,31]. IDH-mutant gliomas in the adolescent may have a higher proportion of non-IDH1R132H mutations (such as IDH1R132G, IDH1R132C, and IDH2R172W), so directed sequencing may be required in selected cases [31]. Also, ependymal or embryonal neoplasms must be ruled-out when a PNET-like histology is present [82]. Diffuse hemispheric high-grade glioma, H3G34-altered should be suspected in any high-grade hemispheric neoplasm negative for IDH1 and 2, lacking OLIG2 and ATRX nuclear staining, and/or with intense nuclear p53 expression [82].

### 3.7. Prognosis

The prognosis is usually bad but better than diffuse midline gliomas, H3K27-altered [2,71]. The median OS is 22 months [64]. The presence of oncogene amplifications is a poor prognostic factor [6,64]. 

The more prevalent methylation of the MGMT promoter (up to 74% of cases in a series of 81 diffuse hemispheric gliomas, H3G34-altered, [64]) and a more accessible tumour location may account for the better survival observed in these patients [6,64]. MGMT promoter methylation status is a positive prognostic factor [64].

### 3.8. WHO Diagnostic Criteria

The current recommended criteria include:

- A diffuse cellular glioma with mitotic activity;
- H3.3 p.G35R (G34R) or p.G35V (G34V) mutation (*H3-3A* [*H3F3A*] c.103G>A, c.103G>C, or c.104G>T);
- Hemispheric location;
- In unresolved lesions, a methylome profile of diffuse hemispheric glioma, H3G34-mutant;
- Desirable criteria include negativity for OLIG2, loss of ATRX expression, and diffuse p53 immunopositivity

## 4. Diffuse Paediatric-Type High-Grade Glioma, H3-Wildtype and IDH-Wildtype

### 4.1. Definition and Grade

Tumours of this family show a diffuse histology with features of malignancy, and are wildtype for histone H3, IDH1, and IDH2. They typically affect adolescents, young adults, and children [4].

They are assigned a WHO grade of 4.

### 4.2. Molecular Pathology

Diffuse paediatric-type high-grade gliomas, H3-wildtype and IDH-wildtype can be further subclassified into three subtypes: RTK1 (Receptor of Tyrosine Kinase-1), RTK2, and MYCN, each with typical molecular alterations. However, as this classification is born from methylome studies, a proportion of cases of each subtype may lack its typical molecular alterations [6,7]:

- RTK1 tumours usually show amplification of PDGFRA (33%). Gliomas arising after radiotherapy show similar molecular features, with mutations in TP53 and amplifications of or mutations in PDGFRA, among other alterations [84,85]. Gliomas arising in the context of Lynch syndrome and constitutive mismatch repair deficiency (CMMRD) typically fall in this category [4].
- RTK2 tumours usually show molecular features of adult-type IDH-wildtype glioblastomas [86], such as the amplification of EGFR (50%), CDKN2A/B homozygous deletions (72%), mutations of the TERT promoter (64%), the gain of chromosome 7 (28%), and the loss of chromosome 10 (50%). However, although key molecular features are similar, pHHGRTK2 and IDH-wildtype glioblastomas are segregated in methylation studies, arguing that these tumours are different.
- MYCN tumours usually show a co-amplification of MYCN (50%) and ID2 (present in 66% of tumours that already have MYCN amplification) [5,14]. CDK4/6 can also be amplified (22%).
- Other alterations include mutations of TP53 (around 50%, and up to 67% in MYCN tumours).
- MGMT promoter methylation is seldom found, more frequently in RTK1 tumours (18%).

### 4.3. Epidemiology

In some paediatric glioblastoma cohorts, this family of tumours shows an incidence around 36% [6,7]. MYCN tumours account for approximately 41% of this category, RTK1 are 38%, while RTK2 tumours are the rarest, at 21% [7]. Most tumours present in children, but adolescents and young adults may also be affected. MYCN patients are younger (median age 8–9 years) compared with RTK1 and RTK2 cases (median age 10–11 years) [7,87].

Most tumours of this family are supratentorial masses with occasional presence in infratentorial areas. MYCN tumours can be found in the brainstem (14%) [5,7,14,87]. RTK1 tumours can be found in the brainstem and cerebellum (18%). Most RTK2 tumours are supratentorial (96%), with extremely rare cases found in the cerebellum [7].

### 4.4. Imaging Features

Radiological features are similar to other high-grade gliomas. MYCN gliomas are more thoroughly described, and usually show a solid mass with necrosis, annular or homogeneous enhancement after contrast infusion, and restriction of diffusion. There may be perilesional oedema and mass effect. Calcification and haemorrhage are not typical features [50,87]. Most cases are hemispherical (>80%), with a preference for temporal lobes [87].

### 4.5. Pathological Features

The morphological features of high-grade gliomas that are mostly seen include brisk mitotic activity, necrosis, and microvascular proliferation [85]. The MYCN subtype has been described in more detail, usually being well-demarcated, hypercellular, undifferentiated neoplasms. A biphasic pattern with spindle and epithelioid cells can be found in MYCN gliomas [50,87]. MYCN gliomas may also have an embryonal PNET-like histology. 

The retention of some glial markers, such as GFAP or OLIG2, is expected, although in undifferentiated cases there may be the loss of both markers. MYCN-altered cases may also express neuronal markers such as NeuN, neurofilaments, and CD56. There may be strong nuclear positivity for p53 [87]. Supratentorial MYCN cases show a loss of PTEN IHC [87]. IDH1, IDH2, and H3 mutations should not be identified. ATRX, H3K27me3, INI1, and BRG1 nuclear staining should be retained [50,87]. 

**Figure 4:**
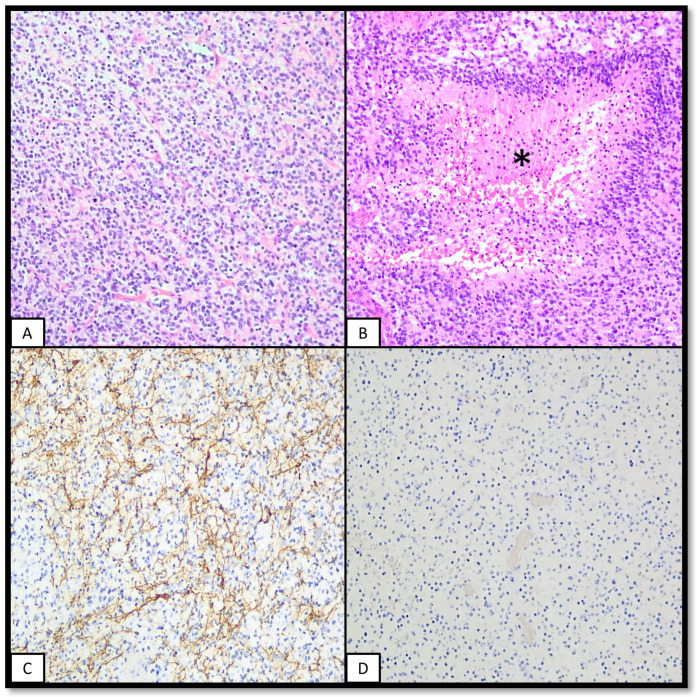
Diffuse paediatric-type high-grade glioma, H3-wildtype and IDH-wildtype. The following figure features a hemispheric neoplasm in a patient with constitutive mismatch repair deficiency. (**A**): A hypercellular glial neoplasm with features of oligodendroglioma (fried egg appearance, plexiform capillary network) is seen. (**B**): Pseudopalisading necrosis (asterisk) was found in some fields. (**C**): IHC for neurofilaments shows a fragmented network, supportive of a diffuse glial neoplasm. (**D**): IHC for PMS2 shows a loss of expression in the tumour and normal cells. IHC for IDH1 and H3K27M were also negative).

Figure 4 features one case of a diffuse paediatric-type high-grade glioma, H3-wildtype and IDH-wildtype.

### 4.6. Differential Diagnosis

The differential diagnosis includes IDH-altered tumours (especially in adolescents) and H3-altered tumours, either H3K27- or H3G34-altered cases, depending on location, epidemiology, and pathological features. The differential diagnosis with H3K27-altered gliomas is especially relevant in MYCN cases, as these tumours can be found in the brainstem. As MYCN gliomas may show a PNET-like morphology, ancillary tests should also cover embryonal neoplasms. IHC staining for H3K27M, H3K27me3, INI1, BRG1, and Lin28Aand FISH for MYCN may be necessary [7,50]. 

### 4.7. Prognosis

Tumours falling in the MYCN category show the worst prognosis, with a median OS of 14 months, comparable to diffuse midline gliomas, H3K27-altered [50]. Pontine MYCN gliomas have a worse prognosis than supratentorial MYCN gliomas, probably due to the location [87]. RTK2 cases show a longer survival (median OS of 44 months), while RTK1 gliomas show an intermediate survival (median OS 21 months) [7]. The histology features of a WHO grade 4 neoplasm are not necessarily correlated with a worse prognosis [8].

Gliomas with a hypermutator phenotype (POLE-mutated, loss of MLH1, CMMRD) tend to be rich in CD8 lymphocytes, and may benefit from checkpoint inhibitor therapy, such as nivolumab [3]. Some case reports of patients with a hypermutator phenotype showed spectacular responses to checkpoint inhibitor therapy [88].

Regarding methylation status of the MGMT promoter, Korshunov et al. reported methylation rates spanning from 0% in the RTK2 subgroup to 18% in the RTK1 subgroup. MYCN-altered cases showed intermediate rates of MGMT methylation in 11% of cases [7].

### 4.8. WHO Diagnostic Criteria

Current recommended criteria include the following:

- A diffuse glioma with mitotic activity occurring in a child or young adult;
- Absence of mutations in IDH1 or IDH2;
- Absence of mutations in H3 genes;
- Methylation profile aligned with pHGG RTK1, pHGG RTK2, or pHGG MYCN, or key molecular features, such as PDGFRA alteration, EGFR alteration, or MYCN amplification;
- Desirable criteria include the presence of microvascular proliferation, necrosis, and the retention of H3K27 trimethylation.

## 5. Infant-Type Hemispheric Glioma

### 5.1. Definition and Grade

This tumour family is defined by a hemispheric location, high-grade astrocytic features, arising in the early childhood (most cases below 1 year), and harbouring Receptor of Tyrosine Kinase (RTK) fusions, including the NTRK family, ROS1, ALK, or MET [4].

No WHO grade is currently assigned.

### 5.2. Molecular Pathology

Most cases are fusion-positive (60–80%) [89,90]. Most fusions identified affect NTRK, ALK, ROS1, or MET [22,89,90,91,92,93,94,95,96,97]. Some cases belonging to this category (per methylome) do not have a fusion in these genes [89]. However, no copy-number changes have been found between fusion-positive and fusion-negative cases. Fusion-negative cases are a puzzling subgroup that needs further investigation [89]. 

### 5.3. Epidemiology

The vast majority of patients are below 1 year of age, most of them younger than 6 months [89,90,91,92,93,94,95,96,97]. Congenital cases have been reported [93,94]. 

### 5.4. Imaging Features

These tumours are usually large neoplasms located in the cerebral hemispheres [90,94]. On CT or MRI scans, a large mass with oedema, hydrocephalus, and mass effect, including herniation, is to be expected [92,95]. Haemorrhage or cystic areas may also be found [92,95].

### 5.5. Pathological Features

Biopsies show a highly cellular astrocytic neoplasm organised in uniform sheets of tumour cells. Mini-gemistocytes, spindle cells, or ganglion-like components can be identified [89]. Most cases show high-grade features including abundant mitotic figures, necrosis, or microvascular proliferation. Most cases are positive for glial markers, like GFAP [91,93,95,97]. 

A proportion of cases may show low-grade histology. It seems that these cases also tend to have ALK alterations [92,94,95,97]. In some instances, a biphasic histology of low- and high-grade areas can be found [97]. Some reported cases showed a lower tumoral grade on follow-up biopsies, raising the possibility of tumour maturation [94,95].

Some tumour case series showed ependymoma-like features, with demarcated morphology, monomorphism, and perivascular pseudorosettes, highlighted with GFAP. OLIG2 IHC was negative. This morphology was associated with ALK rearrangements demonstrated by IHC and FISH [96]. 

Rare cases may be less cellular. Mineralisation, calcification, or xanthomatous changes may be observed [89]. Fusion-negative cases may proliferate less that fusion-positive ones [89].

### 5.6. Differential Diagnosis

The differential diagnosis will depend on patient age, location, and morphology. The most frequent types of infant brain tumours include a wide range of histologies, such as medulloblastoma, ependymoma, and low-grade gliomas, also including pilocytic astrocytoma and desmoplastic infantile ganglioglioma/astrocytoma [89,98]. 

The presence of hypercellularity and high-grade features may require ruling out ependymoma and embryonal tumours. On the other hand, a lack of high-grade features will require discarding other low-grade neoplasms. 

A high-grade glial neoplasm in a very young patient (less than 1 year old) should raise suspicion and prompt molecular studies to rule out RTK fusions. Depending on the context (age, location, radiology, and histology), other alterations should also be assessed (H3 mutations, BRAF alterations). 

Regarding NTRK rearrangements, not all NTRK fusions are restricted to this family of tumours. NTRK rearrangements may be identified in paediatric and adult patients in a wide range of histologies, including gliomas, glioneuronal neoplasms, and embryonal tumours [99]. NTRK fusions may also appear as subclonal alterations in otherwise typical neoplasms, suggesting that these may occur as secondary alterations [99]. 

### 5.7. Prognosis

Most of these tumours have a better prognosis than other paediatric or adult HGGs, even without complete resection or radiation therapy [89,94,98,100]. Most treatment courses follow chemotherapy with a delay or no radiotherapy due to neurodevelopmental adverse effects [90,98,100]. Due to the large tumour size, small circulating blood volume, and comorbidity, gross total resection may not be achieved in all patients [90,98].

Even in relapses or refractory disease to conventional chemotherapy, the availability of specific Tyrosine Kinase inhibitors (crizotinib, entrectinib, and larotrectinib) remains a promising line of treatment, with favourable outcomes in several reported cases [89,90,91,94]. Similar encouraging results are found in other fusion-positive solid neoplasms [101]. Fusion-negative cases lack this therapeutic option and bear a worse prognosis under standard treatments [89].

This family of tumours remains a paradigm of potential candidates to benefit from precision medicine. However, more investigation efforts must be made regarding the cryptic fusion-negative infant-type hemispheric glioma.

### 5.8. WHO Diagnostic Criteria

The current recommended criteria include the following:

- Cellular astrocytoma;
- Presentation in early childhood;
- Cerebral hemispheric location;
- Presence of a typical Receptor of Tyrosine Kinase abnormality or methylation profile aligned with infant-type hemispheric glioma

## 6. Conclusions

Many entities converge under the pHGG category, an umbrella term that comprises a highly heterogeneous family of tumours that can bear similar histological features. The main features of pHGGs are summarised in Table 1. Thanks to molecular pathology, pHGGs can be better diagnosed and classified, and some of them may benefit from targeted therapies. However, although molecular pathology is an essential part of the diagnostic pathology of gliomas, morphological evaluation is still a necessary element of the diagnostic process, acting as a foundation for adequate molecular assessment.

Also, with constant updates in diagnostic categories and new molecular markers, published literature inevitably needs to be updated. Revision and actualisation of historic cohorts with current diagnostic schemes would yield invaluable information to better understand the pathology of pHGG.

Finally, although much has been achieved in a few years, it is true that we still know little of pHGG. Even though it can be demoralising that knowledge is never “complete”, is this imperfection that allows us to keep pursuing expertise, for new lines of investigation to be initiated and new promising therapeutic schemes to be attempted. With this eagerness to know, in conjunction with multidisciplinary research efforts, what today are incurable diseases, maybe tomorrow will become curable, as has already happened with some other malignancies [4].

## Figures and Tables

**Table 1:** Main Features of Paediatric High-Grade Glioma.

	Diffuse Midline Glioma H3K27-Altered	Diffuse Hemispheric Glioma, H3G34-Altered	Diffuse Paediatric-Type High-Grade Glioma, H3-Wildtype and IDH-Wildtype	Infant-Type Hemispheric Glioma
Driver Molecular Events	Loss of H3 trimethylation - H3.1 or H3.3 K27 point mutations (H3K27M, H3K27I) - Overexpression of EZHIP - Mutations in EGFR (bithalamic glioma)	H3.3 G34R or G34V point mutations - G34R is more common (94%) than G34V (6%) - Usually mutations in ATRX (95%) and TP53 (88%) - Most cases show methylation of MGMT promoter	IDH and H3 Wildtype Methylation subtypes: - RTK1 (38%): Amplification of PDGFRA (33%). Also in CMMRD and Lynch syndrome, or after radiotherapy - RTK2 (21%): Similar to IDH-wildtype glioblastomas: EGFR amplification (50%), CDKN2A homozygous deletion (72%), TERT promoter mutations (64%), +7 (28%), −10 (50%) - MYCN (41%): MYCN amplification (50%), usually coamplified with ID2	May harbour fusions with NTRK, ALK, ROS1 or MET
CNS WHO grade	Grade 4	Grade 4	Grade 4	Not assigned
Epidemiology	Most patients are children and adolescents H3.1-altered cases are younger than H3.3-altered cases (which may affect adults)	Most are adolescents and young adults Usually from 11 to 30 years	MYCN cases show a median age of 8–9 years RTK1 and RTK2 are older, with a median age of 10–11 years	Most cases below 1 year of age May be congenital
Location	Midline structures - H3.1-altered cases restricted to the pons - H3.3-altered cases distributed along the midline (thalamus, pons, spinal cord, etc)	Hemispheric, with preference for temporal and parietal lobes	Most cases are supratentorial masses RTK1 (18%) or MYCN (14%) tumours may appear in the brainstem (both) or cerebellum (RTK1)	Cerebral hemispheres
Histopathology	Diffuse pattern of infiltration Usually astrocytic features High-grade features may not be present	Two patterns, usually with high-grade features: - Glioblastoma multiforme-like with glial features - PNET-like, reminiscent of embryonal tumours	Presence of high-grade features May be circumscribed May show biphasic pattern (spindle and epithelioid) or PNET-like histology	Hypercellular, usually high-grade features
IHC	H3K27me3 loss is essential in all subtypes H3K27M or EZHIP positivity depends on the subtype Most cases are GFAP+, OLIG2+, and MAP2+ Bithalamic gliomas are usually GFAP+, OLIG2-, and MAP2-	Usually GFAP+ and OLIG2- Loss of ATRX IHC and diffuse p53 staining are typical PNET-like cases may stain with neuronal markers IHC for H3G34R or H3G34V may be of help	Glial markers are usually positive (GFAP+, OLIG2+) MYCN cases may express neuronal markers (NeuN, Neurofilaments, CD56) H3KK27me3 should be retained IDH1 and H3K27M must be negative	Usually positive for glial markers ALK rearranged cases may show IHC positivity
Differential Diagnosis	Consider DMG H3K27-altered in midline tumours pHGG H3- and IDH-wildtype, MYCN subtype, may also affect the midline (consider FISH for MYCN) Consider low-grade tumours if high-grade features are absent	Essential to prove presence of an H3G34 point mutation in hemispheric neoplasms Consider IDH-altered gliomas in adolescents and adults If PNET-like histology, also consider embryonal or ependymal neoplasms	Dependent on location and morphology Essentially excluding H3-altered or IDH-altered gliomas PNET-like histology may require differential diagnosis with embryonal neoplasms	Dependent on morphology May include embryonal neoplasms (medulloblastoma), ependymomas, or gliomas Testing for RTK fusions is useful for diagnosis
Prognosis	Dismal, median OS of 11 months Longer survival in H3.1-altered cases (median OS 15 months) Bithalamic glioma has equally bad prognosis (median OS of 8 to 12 months)	Bad prognosis, but better (median OS 22 months) than DMG H3K27-altered Methylation of MGMT promoter may be a factor in survival	Survival is bad, but depends on methylation subtypes: MYCN subtype is comparable to DMG H3K27-altered (median OS: 14 months); RTK1 and RTK2 show longer survival (median OS of 21 and 44 months)	Better than other pHGGs Consider Tyrosine Kinase inhibition if possible

## Data Availability

No new data were created or analysed in this study. Data sharing is not applicable to this article. Figures are original and represent key features of each type of glioma commented on the text, and are equivalent to data published by other authors on this matter.
